# Degraded Sericin Significantly Regulates Blood Glucose Levels and Improves Impaired Liver Function in T2D Rats by Reducing Oxidative Stress

**DOI:** 10.3390/biom11081255

**Published:** 2021-08-23

**Authors:** Heng-Da Wang, Zhi-Hao Zhong, Yu-Jie Weng, Zhen-Zhen Wei, Yu-Qing Zhang

**Affiliations:** School of Biology and Basic Medical Sciences, Medical College, Soochow University, Suzhou 215123, China; 20194221049@stu.suda.edu.cn (H.-D.W.); 20194221048@stu.suda.edu.cn (Z.-H.Z.); annieweng1010@163.com (Y.-J.W.); weizz112@163.com (Z.-Z.W.)

**Keywords:** sericin, oral administration, T2D, rats, hypoglycemic, oxidative stress, liver damage

## Abstract

Sericin could be degraded well into low-molecular-weight sericin (SS) through a novel and environmentally friendly recycling process using an ultrasonically degumming method in Ca(OH)_2_ aqueous solution. The oral administration of the SS has an evidently hypoglycemic effect on STZ-induced T2D rats. At oral doses of 2.5 and 5% SS for four weeks, the fasting blood glucose decreased by over 60% compared with that in the untreated model group. Oral glucose tolerance and insulin tolerance were ameliorated by the peptide treatment. The serum insulin level was reduced by approximately 35%, the insulin resistance index was reduced by more than 66%. The 8-hydroxy-2 deoxyguanosine level showed a large reduction of 20%, and the total antioxidant activities significantly increased. Hematoxylin-eosin staining and fluorescent immunostaining sections showed that liver and pancreas damage was partly recovered in T2D rats. In summary, oral SS demonstrated evidently hypoglycemic effects mainly related to reducing oxidative stress in the damaged liver and pancreas of T2D rats. Therefore, these results have suggested that the degraded sericin has a potential use in SS-based healthy functional food or hypoglycemic drugs as a waste recovered from sericulture resources.

## Highlights

1. An innovatively cleaner technology by Ca(OH)_2_ as degumming reagent can recover a large amount of the degraded sericin peptides and avoid the environmental pollution of the alkaline wastewater from traditional degumming process.
2. Oral sericin significantly improves the symptoms of hyperglycemia and liver function in T2D rats by reducing the level of oxidative stress.
3. Sericin from silk industrial waste has a potential use in biologically hypoglycemic drugs or healthy food additive with hypoglycemic function.

## 1. Introduction

The sericulture industry has been an important economic industry since ancient China. Since 2010, the annual silkworm cocoon production in China has exceeded 6 million tons, which accounts for approximately 70% of total production worldwide. In the traditional silk-processing industry, over 40,000 tons of sericin are discharged with alkaline wastewater during the degumming process every year. Discarded sericin consumes large quantities of oxygen during the degradation process, which can eventually result in large quantities of environmental and water resource pollution [1,2]. Therefore, the development of a new clean-processing technology for sericin is necessary, which might lead to the development of new sericin uses and reduce the environmental pollution associated with the current sericin degumming and degradation processes [3].

Sericin is a type of natural macromolecular protein found on the surface of silk fibroin and is typically separated from silk fibroin during silk production processes. Currently, the separation and purification methods used for sericin separation primarily include: high-temperature hydrolysis [4], high-temperature alkaline salt water hydrolysis [5], acid extraction [6], protease degumming [7](Arami et al., 2010), neutral soap degumming [8], high-temperature and high-pressure degumming [9], and other degumming methods. Our group found that the use of a Ca(OH)_2_ aqueous solution can completely remove sericin from silk fibroin with no significant impact on the physical properties of the degummed silk fibroin. Moreover, Ca(OH)_2_ can easily be precipitated from the degummed solution as CaSO_4_ through an acid-base neutralization reaction. After separation, the calcium salt can be used as an inorganic fertilizer for plants or as building fillers without causing new pollution [10]. By comparing the physical and chemical properties of the sericin obtained using different methods, we found that the sericin obtained through the Ca(OH)_2_ degumming method has lower molecular weights, typically under 30 kDa, and was superior in antioxidant capacity, ultraviolet (UV) resistance, and α-glycosidase inhibitory activities than sericin obtained using other methods in vitro [11].

In recent years, researchers have discovered that sericin has antibacterial (Xue et al., 2016), anti-UV, antioxidation [12], whitening [13], immune enhancement [14], and wound healing promotion [15] properties. In addition, sericin has the effects of lowering blood lipids [16,17], anti-Alzheimer’s disease [18], anti-tumor, and other biological activities in vitro. Lee et al., detailed that less than 1 kDa of sericin peptides had better hypoglycemic effects than high-molecular-weight sericin peptides in vitro [19]. The high-fat diet-fed containing sericin peptides demonstrated reduced blood lipid levels, improved glucose tolerance, and increased serum adiponectin levels compared with untreated high-fat diet-fed rats [20]. The sericin extracted from a boiled solution of colored cocoons enhances the insulin PI3K/AKT signaling pathway in the liver of a T2D rat [21], reduced the destruction of pancreatic islet cells, protected kidney and other organs [22], improved insulin resistance [23], promoted corneal wound healing [24], improved the observed disorder in the growth hormone/insulin-like growth factor 1 axis, and reduced hippocampal damage in T2D rats [25]. In addition, sericin has been shown to reduce liver steatosis, allowing zymogen granules to preserve extracted liver mitochondria and decrease active oxygen production [26].

Unfortunately, most of the sericin extracts in the above-mentioned studies are derived from colored cocoons of silkworm. However, these sericin extracts are typically rich in β-carotene, flavonoids, and polyphenols, which are known to exert superior antioxidant, antibacterial, and hypolipidemic activities. Therefore, the observed biological effects, especially the lowering of blood sugar, may be related to the presence of the above-mentioned active molecules. In this study, the degraded sericin peptides from the white cocoons without above active substances, produced in large quantities in sericulture, were used as the oral experimental materials for STZ-induced T2D rats. The antioxidation and hypoglycemic activities of the low-molecular-weight sericin peptides were investigated in detail in T2D rats.

## 2. Materials and Methods

### 2.1. Preparation of Sericin Peptides and Rat Feed

A certain amount of white cocoon shells or waste silk from silkworm *Bombyx mori* (Sohao×ZhongYe), purchased from Nantong, Jiangsu) was weighed and degummed twice with 0.025% Ca(OH)_2_ solution (*w*/*w*: 1:90) at 90 °C for 2 h. The degumming liquid was neutralized to pH 7.0 by the addition of 1 mol/L sulfuric acid, and the solution was concentrated by rotary evaporation. After being centrifuged at 10,000 rpm for 30 min, insoluble impurities were removed [10,11]. The supernatant was collected and dried to prepare low-molecular-weight sericin powder.

The irradiated rat feed was crushed, and the Ca(OH)_2-_treated sericin peptide powder was mixed into the ordinary feed powder at ratios of 1, 2.5, and 5% to prepare different doses of SS feed.

### 2.2. Animal Testing

Seventy specific-pathogen-free (SPF) male Sprague-Dawley (SD) rats (weighing 150–200 g) were obtained from and raised in the Experimental Animal Center of Soochow University. All rats were allowed free access to food and water and were raised under standard conditions with controlled humidity (50–80%) and temperature (18–25 °C) with a 12-h light/dark cycle.

After adaptation for one week, all rats were randomly divided into two groups: a normal group (*n* = 10) and a T2D group (*n* = 40). The normal rats continued to be fed with normal feed, and a T2D was induced in the T2D group through the following steps. After being fasted overnight, with free access to water, and a low dose [35 mg/kg·body weight (BW)] of streptozocin (STZ) was injected into each animal for three consecutive days. STZ was dissolved in a 0.1 mmol/L sterile citric acid buffer at pH 4.4 at 4 °C. After injection, the rats were fed with normal feed. Seven days after the first injection, rats were fasted for 10 h, and fasting blood glucose (FBG) levels were measured using the tail vein blood test. Rats with FBG levels above 11.1 mmol/L were considered to represent successful T2D animals.

The successfully modeled rats in the T2D group were randomly divided into five groups, containing eight rats in each group: a model group, a 0.5% metformin group (PC group), and low, medium, and high dose SS groups (LSS, MSS, and HSS groups). After grouping, the rats in the normal and model groups were fed with normal feed, and the rats in the PC, LSS, MSS, and HSS groups were fed a mixed diet containing 0.5% metformin, 1, 2.5, and 5% sericin, respectively. The treatment was administered for four weeks, and the BWs and FBG levels of the rats were monitored every week.

Four weeks later, after being fasted overnight, blood was collected from the eyeball, and all rats were sacrificed by cervical dislocation. The blood was maintained at room temperature for 2 h and then centrifuged. The supernatant was saved for testing. The liver and pancreas tissues were weighed, and portions of each tissue type were fixed in 4% formalin. The remaining tissue was stored at −80 °C for further testing. All animal tests were performed in accordance with the relevant regulations and provisions required by the International Animal Welfare Committee and the Animal Test Operations and Ethics Committee of Soochow University (Approval Code: 201911A063; Approval Date: 5 November 2019).

### 2.3. Measuring BWs and FBG Levels

During the test period, the body weight and fasting blood glucose (FBG) of each rat were measured weekly. The rats were fasted for 10 h before blood collection, during which time drinking water was not prohibited, and they were changed to clean cages before eating. Firstly, the fasting body weight of each rat was measured; the blood was collected by tail-cutting method, and the FBG was measured with a blood glucose meter (ONETOUCH, Johnson).

### 2.4. Oral Glucose Tolerance and Insulin Tolerance Measurement

On the 18th day of treatment, an oral glucose tolerance test (OGTT) was performed to measure the blood glucose levels of rats. The initial blood glucose value (0 min) was measured after fasting for 12 h. Then, 1.5 g/kg·BW glucose was administered via gavage, and blood glucose levels were measured at appropriate time intervals (30, 60, 90, and 120 min) by the tail trimming method.

On the 22nd day of treatment, all rats in each group underwent an insulin tolerance test (ITT). The initial blood glucose level (0 min) was measured after fasting for 6 h. Then, 0.5 U/kg·BW of insulin was injected intraperitoneally, and the following blood glucose levels of the rats were measured at appropriate time intervals (30, 60, 90, and 120 min) by the tail trimming method.

The OGTT and ITT curves are made using these time intervals (0, 30, 60, 90, and 120 min) as the abscissa and the corresponding blood glucose value as the ordinate.

### 2.5. Fasting Serum Insulin Detection and Homeostasis Model Assessment of the Insulin Resistance Index and Insulin Sensitivity Index

Serum insulin levels were evaluated using Insulin Assay kit (purchased from Nanjing Jiancheng Co., Ltd., Nanjing, China).

The Homeostatic Model Assessment for Insulin Resistance (HOMA-IR) was calculated using the following formula: [fasting blood glucose value (mmol/L) × fasting serum insulin value (mIU/L)]/22.5. The insulin sensitivity index (ISI) was calculated using the following formula: Ln [1/(fasting blood glucose value × fasting serum insulin value)].

### 2.6. Determination of Glycated Serum Protein Levels

The levels of glycated serum proteins (GSPs) in the rats from each group were measured using a GSPs Assay kit (purchased from Nanjing Jiancheng Co., Ltd.). The GSP test was carried out according to the product manual and the method reported by Schleicher and Wieland [27].

### 2.7. Detection of Six Blood Lipid and Hepatic Function Parameters

The levels of alanine aminotransferase (ALT), aspartate aminotransferase (AST), triglycerides (TG), high-density lipoprotein cholesterol (HDL-C), low-density lipoprotein cholesterol (LDL-C), lactate dehydrogenase (LDH), and non-esterified free fatty acids (NEAF1) were all measured with the Abbott Architect c8000 clinical chemistry analyzer.

### 2.8. Liver Weight and Organ Body Coefficients

After the rats were sacrificed by cervical dislocation, the livers were removed and weighed. Organ coefficients were calculated as the ratio of tissue weight (wet weight, g) to BW (g).

### 2.9. Hepatic Biochemical Assay

The hepatic 8-hydroxydeoxyguanosine (8-OHdG) levels were determined by 8-OHdG assay kit (purchased from Greenleaves Biological Co., Ltd. Shanghai, China). The hepatic glycogen levels were determined with a hepatic glycogen assay kit (purchased from Greenleaves Co., Ltd.). The levels of total superoxide dismutase (T-SOD), glutathione peroxidase (GSH-PX) activity, malondialdehyde (MDA), and total antioxidant capacity (T-AOC) were determined by commercially available kits. Finally, the protein concentration of the liver tissue was determined by the BCA method (all purchased from Nanjing Jiancheng Co., Ltd.).

### 2.10. Preparation of Histopathological Sections

Pancreas and liver tissues were fixed in formalin followed by dehydrated with a gradient alcohol series, made transparent in xylene, embedded in paraffin, and sectioned on a paraffin microtome at a slice thickness of approximately 3 µm. The slices were stained with hematoxylin-eosin (HE) and sealed with neutral gum. Histopathological changes were observed under an optical microscope, and images were obtained.

### 2.11. Immunofluorescence Staining

To remove wax, the slides were first heated to 65 °C for 30 min, successively followed by dewaxing with xylene I and xylene II for 15 min each. The deparaffinized slides were then dehydrated using a gradient alcohol series and rinsed with tap water for 10 min. High-pressure antigen retrieval was performed using a 0.01 mol/L sodium citrate buffer solution for 15 min. After cooling down, the slides were washed 3 times, with 0.02 mol/L phosphate-buffered saline (PBS). Ethylenediaminetetraacetic acid (EDTA, pH 8.0) was added to boiling water, covered with a stainless steel pot lid; however, the lid cannot be locked. The slides were placed in a metal staining rack, slowly pressurized, and soaked in the buffer for 5 min. Then, the lid was locked, and the small valve would rise. After heated for 10 min, the slides were placed in cold water, and the lid was opened when the small valve fell. The slides were incubated with primary antibody at 4 °C overnight and then rinsed 3 times with 0.02 mol/L PBS. The insulin antibody was diluted at an appropriate ratio. The slides were incubated in secondary antibody at room temperature for 1 h. The slides were rinsed 3 times × 3 min each in 0.02 mol/L PBS. Anti-quenching mounting tablets and 4′,6-diamidino-2-phenylindole (DAPI, 1:500) were used to mount the slides, and slides were stored at −20 °C in a refrigerator until imaged using a fluorescence microscope.

### 2.12. Statistical Analysis

The data obtained during the experiment were processed by Graph Prism 8 statistical software. The results are presented as the mean ± standard deviation. Group data were compared using a one-way analysis of variance (ANOVA) analysis. A *p*-value of *p* < 0.05 was considered to be significant.

## 3. Results and Analysis

### 3.1. Body Weight Measurements

**Figure 1:**
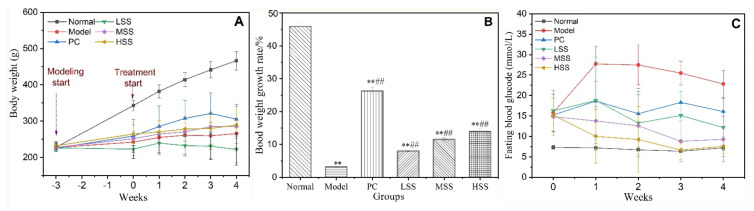
Effects of oral SS administration on the body weights and FBG level of T2D rats. (**A**) Body weights; (**B**) body weight growth rate; (**C**) FBG levels. Normal: normal group; Model: diabetic model group; PC: 0.5% metformin administration; LSS, MSS, and HSS: 1%, 2.5, and 5% SS administration, respectively; *n* = 10, ** *p* < 0.01, vs. normal group; ^##^ *p* < 0.01, vs. model group.

Weight loss is a critical feature of diabetes. The BWs of rats were monitored weekly after successful modeling was established. Changes in BW for each group are shown in Figure 1A, and the BW growth rates were shown in Figure 1B. The BW of normal group increased to 500 ± 27 g. The BW of model group showed a slight increase during the first week, then decreased in subsequent weeks until the final BW was similar to the initial BW (250 ± 39 g). The BW of the PC group gradually increased during the first three weeks, at a BW growth rate of 26.25%, which was significantly different from that for model group (*p* < 0.05). The BWs of the MSS and HSS groups were higher than that of the LSS groups, with no significant difference observed between MSS and HSS groups. The BW of the LSS group increased slightly during the first week before decreasing during the following three weeks. The BWs of both the MSS and HSS groups showed moderately increasing trends, which were significantly increased compared with the BW of model group at each time point (*p* < 0.05). The BW growth rates of three SS groups increased by 7.9, 11.5, and 13.9%, respectively. The medium and high doses of oral SS treatment demonstrated preventive effects against weight loss of diabetic model rats.

### 3.2. FBG Levels

FBG is an important indicator for determining the severity of diabetes. The effectiveness of diabetes medication is first reflected by a reduction in blood glucose levels. The blood glucose levels for each group during each week are shown in Figure 1C. The blood glucose levels for the normal group were approximately 7 mmol/L every week. In contrast, the FBG level of the model group was maintained at a high level (≥23 mmol/L), which was significantly higher than the levels for all other groups (*p* < 0.01). The FBG level of the PC group fluctuated and remained near 15 mmol/L, which represented a significant reduction of 34% during the fourth week compared with the model group. During the four weeks of oral SS administration, the FBG levels of the rats in three SS groups showed a downward trend overall. The FBG levels of the LSS group showed a slight downward trend, decreasing to 12.1 ± 5 mmol/L by the fourth week. The FBG levels of the rats in the MSS and HSS groups were also significantly decreased. Two weeks after the oral administration of sericin peptides, the FBG levels for the MSS and HSS groups were 8.5 mmol/L and 7.5 mmol/L, respectively, which remained stable during the last two weeks. Compared with the final FBG level of model group, the final FBG levels of three SS groups decreased by 46.9, 62.3, and 67.1%, respectively. The results indicated that oral SS treatment could inhibit abnormal blood glucose increase in diabetic rats induced by STZ with obvious dose effect.

### 3.3. Oral Glucose Tolerance Test Results

**Figure 2:**
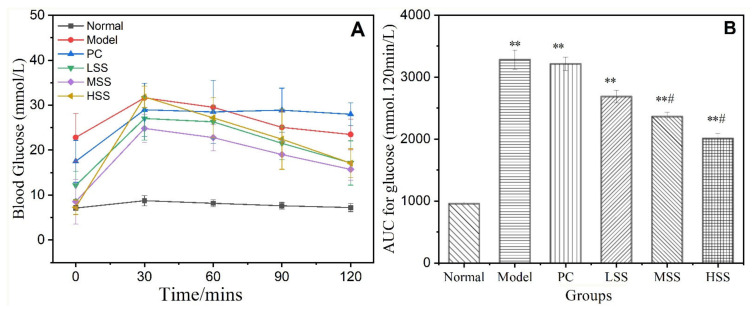
Effects of oral SS administration on the OGTT results of T2D rats. (**A**) Blood glucose; (**B**) AUC for blood glucose. Normal: normal group; Model: diabetic model group; PC: 0.5% metformin administration; LSS, MSS, and HSS: 1, 2.5, and 5% SS administration, respectively. *n* = 3, ** *p* < 0.01, vs. normal group; ^#^ *p* < 0.05, vs. model group.

The OGTT reflects the insulin-dominant effects on the blood glucose level, which can be determined by observing the rate of decrease in blood glucose levels following the bolus delivery of glucose. It will drop under 7.8 mmol/L after 2 h for a normal OGTT result. A blood glucose level that remains above 11.1 mmol/L after 2 h indicates the presence of diabetes. The OGTT results are shown in Figure 2. The blood glucose level of normal group remained relatively stable at approximately 7.5 ± 0.4 mmol/L. The blood glucose levels of the T2D rats became significantly enhanced and peaked after 30 min before gradually decreasing during the following 90 min. The blood glucose levels of model group showed a downward trend within 30–120 min, but the decline rate was significantly slowed. The blood glucose level in model group remained at a high level (>23 mmol/L), and the AUC (the area under the blood glucose curve) was three times that of normal group. The blood glucose levels and AUC values for the PC group were similar to that for the model group, indicating that in this experiment, low-dose oral metformin administration had no significant effect on abnormal oral glucose tolerance. Four weeks after oral SS administration, the blood glucose levels in all SS groups significantly decreased, and the blood glucose levels during the last 30 min of the OGTT were significantly reduced compared with that for the model group (*p* < 0.05). At 120 min, the blood glucose levels in all three SS groups were approximately 16 mmol/L, and the AUC values gradually decreased with increasing SS concentrations. The blood glucose decline rates of rats in three SS groups were 36.6, 36.7, and 46.3%, respectively. The AUC values and blood glucose decline rates for the SS groups appeared to be dose-dependent. These results implied that oral SS administration might promote insulin secretion in diabetic model rats, enhance glucose utilization, and reduce blood glucose levels by improving the abnormal glucose tolerance of T2D rats.

### 3.4. Insulin Tolerance Test Results

**Figure 3:**
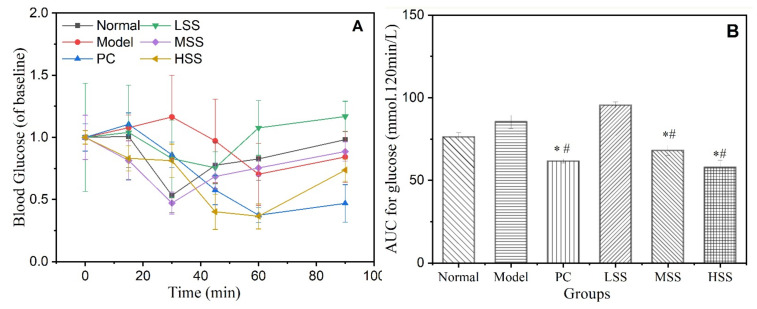
Effect of oral SS administration on the ITT results of T2D rats. (**A**) ITT results, with baseline blood glucose levels set to 100%; (**B**) AUC of ITT. Normal: normal group; Model: diabetic model group; PC: 0.5% metformin administration; LSS, MSS, and HSS: 1, 2.5, and 5% SS administration. *n* = 3, * *p* < 0.05, vs. normal group; # *p* < 0.05, vs. model group.

The ITT evaluates the body’s ability to withstand insulin., which reflects the fragility of the glucose metabolism balance. ITT tests the responsiveness and comprehensive efficacy of glucocorticoids by observing the grade and speed of declines in the blood glucose level. Blood glucose levels of ITT, using the initial blood glucose value as the baseline value, are shown in Figure 3A, and the AUC values for the ITT were calculated, as shown in Figure 3B. The blood glucose level of each group first decreased and then increased within 90 min after the insulin injection. However, the blood glucose levels in each group did not reach the minimum values at the same time. The minimum values for the normal, MSS, and HSS groups appeared at 30 min, whereas the LSS group achieved a minimum value after 45 min and the model and PC groups did not achieve a minimum value until 60 min. These different recovery times may be associated with the insulin reaction time. Oral SS administration could effectively shorten the insulin reaction time, with a dose-dependent effect. The blood glucose level of normal group dropped to 52.9% of the initial value but eventually returned to the initial value. In the model group, the blood glucose level only decreased by 29.6%, but only recovered to 84.1% of the initial value. The AUC of model group also increased significantly (*p* < 0.05) compared with that of the normal group. The blood glucose level of the PC group was significantly reduced to 37.4% of the initial value, and the blood glucose level was only restored to 70.3% after 90 min. The AUC value decreased significantly, indicating that metformin could effectively improve the dominant insulin efficacy of T2D rats, but did not enhance insulin responsiveness. The blood glucose levels of three SS groups were reduced to 75, 44, and 36% of their respective initial values and were restored to 126, 89.5, and 79.3% of their respective initial blood glucose values at 90 min. Therefore, oral SS administration might enhance hypoglycemic responses and contribute to the maintenance of blood glucose stability. The AUC values for three SS groups showed a dose-dependent decreasing trend, which indicated that SS could significantly enhance the dominant effects of insulin and improve insulin sensitivity, with good dose effects. The above results suggested that that oral sericin administration for four weeks could effectively reduce insulin tolerance and improve insulin sensitivity in T2D rats, especially in the MSS and HSS groups.

### 3.5. Serum Insulin Levels

**Figure 4:**
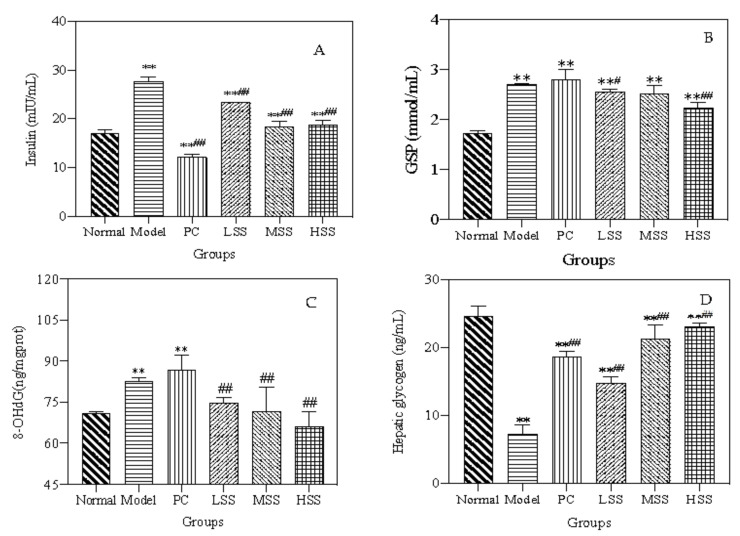
Effects of oral SS administration on physiological indexes of T2D rats. (**A**) Insulin; (**B**) GSP level; (**C**) 8-OHdG level; (**D**) Hepatic glycogen. Normal: normal group; Model: diabetic model group; PC: 0.5% metformin administration; LSS, MSS, and HSS: 1, 2.5, and 5% SS administration; *n* = 3, ** *p* < 0.01, vs. normal group; ^##^ *p* < 0.01, vs. diabetic model group.

When blood glucose levels increase, insulin is compensatorily secreted, and the body’s serum insulin levels often exceed the normal level. To demonstrate the effect of oral sericin administration on insulin resistance in T2D rats, the serum insulin levels were measured in each group. As shown in Figure 4A, the serum insulin level was 17 ± 0.7 mIU/L in normal group, whereas the value in model group was 1.78 times higher, at 27.6 ± 1.1 mIU/L (*p* < 0.01). The serum insulin level of the PC group was significantly decreased compared with both the model and normal groups, indicating that oral metformin administration was able to improve abnormal insulin secretion in T2D rats. After four weeks of treatment, the serum insulin level of the PC group was significantly lower than that of model group. The insulin level of the LSS group was reduced by 15.4% compared with model group, and the serum insulin levels of the MSS and HSS groups were similar, with both at approximately 18 mIU/L. This level was approximately 35% lower than that in model group and was similar to the normal insulin level. These test results suggested that oral sericin administration, especially the middle- and high-dose groups, could significantly reduce the insulin levels in T2D rats with a positive dose-dependent effect.

### 3.6. Homeostasis Model Assessment of IRI and ISI

HOMA-IR is used to evaluate the compensatory oversecretion of insulin for the maintenance of blood glucose stability when the effects of insulin on glucose ingestion and utilization declines [28]. The ISI is used to describe the degree of insulin resistance [29]. Lower ISI values are associated with the reduced effectiveness of a single unit of insulin. Table 1 presents the HOMA-IR and ISI values for all groups. The HOMA-IR value of model group was significantly higher than that for normal group (*p* < 0.001). The HOMA-IR value of the PC group and three SS groups all decreased significantly compared with that of model group (*p* < 0.001). The HOMA-IR values of three SS dose groups decreased by 50, 66, and 70%, respectively. Compared with the normal group, the ISI value of the model group was significantly reduced by 32.7% (*p* < 0.001). The ISI value of the PC group was significantly higher than that of the model group. Compared with model group, the ISI values of three SS groups increased by 10, 14, and 15%. These results indicated that oral SS administration could effectively reduce insulin resistance and enhance insulin sensitivity, with a significant dose-dependent effect. Therefore, oral SS administration could improve the ability of insulin to lower blood glucose levels and reduce the oversecretion of insulin to maintain blood glucose stability. Oral SS administration could improve the unit insulin effect, promote the decomposition of glucose, and urination to reduce blood glucose levels.

### 3.7. Glycated Serum Protein Levels

Glycated serum albumin is a polymer ketamine structure formed by a non-enzymatic glycation reaction. GSP levels can reflect the average blood glucose level over the previous two weeks and play an important role in judging the ability to control blood glucose levels in T2D patients. As shown in Figure 4B, the GSP level in model group was 2.71 ± 0.01 mmol/L, which was 1.5 times higher than that in the normal group. The GSP level of the PC group was 2.91 ± 0.01 mmol/L, which was slightly higher than the GSP level of model group. After oral SS administration, the GSP levels of three SS groups decreased by 0.1, 0.2, and 0.3 mmol/L, which were significantly lower than that of model group (*p* < 0.05, *p* < *0.01*). These results indicated that oral SS administration could reduce the GSP levels of T2D rats with a dose-dependent effect by lowering blood glucose levels.

### 3.8. Hepatic Function Assessment

Type 2 diabetes is often accompanied by liver cirrhosis and abnormal liver function, which can further aggravate diabetic symptoms. To explore the effects of SS on hepatic function of T2D rats. The activities of six serum liver function enzymes were measured in all groups, including AST, ALT, total protein (TP), albumin (ALB), total bilirubin (TBIL), and alkaline phosphatase (ALP), as shown in Table 2.

The ALT and AST levels reflect the liver injury sensitivity index. ALT is primarily distributed in the liver cytoplasm, and increased serum ALT levels reflect damage to the liver cell membrane. Similarly, AST is primarily distributed in the liver cytoplasm and liver cell mitochondria [29], and increased serum AST levels indicate that liver cells are damaged to the level of the organelles. When liver damage is severe, the AST/ALT ratio will increase significantly. The serum ALT and AST levels in the model group were significantly enhanced compared with those in the normal group (*p* < 0.05, *p* < 0.01), and the AST/ALT ratio was greatly increased compared with those in the normal group. The ALT and AST levels in T2D rats were severely affected, indicating abnormal liver function. After oral SS administration, the serum ALT level of LSS group rats was higher than that for model group, whereas the serum ALT levels of the MSS and HSS groups decreased compared with that in the model group, with a particularly notable decrease observed for the HSS group (*p* < 0.05). The ALT level for the HSS group was similar to that for the normal group. These results indicated that SS could inhibit the increase in serum ALT levels associated with T2D. The serum AST levels of three SS groups significantly decreased compared with that in the model group (*p* < 0.01), to levels even below that of normal group. The AST/ALT ratios for three SS groups were all approximately 2.5, which was half that for the model group. These results indicated that oral SS administration could inhibit the increase in serum ALT and AST and prevent the liver damage caused by diabetes to a certain extent.

The TP and ALB levels reflect the ability of the liver to synthesize proteins. ALB plays important roles in the maintenance of blood osmotic pressure, the transport of metabolites, and nutrition. The TP and ALB levels in model group increased significantly compared with those in the normal group (*p* < 0.05, *p* < 0.01), and the TP levels of the MSS and HSS groups decreased by 9 and 5% (*p* < 0.01), respectively, resembling the normal level. After oral SS administration, the ALB level of three SS groups decreased by 5.24 G/L, 6.24 G/L, and 4.51 G/L compared with model group (*p* < 0.01). These results indicated that oral SS administration, especially at medium and high doses, could reduce the TP and ALB levels and gradually restore the synthetic function of the liver in T2D rats.

The TBIL and ALP levels can also be used to judge the degeneration and necrosis of liver cells. When bilirubin metabolism is impaired, or intrahepatic cholestasis occurs, the TBIL and ALP levels will increase abnormally. As shown in Table 2, the TBIL and ALP levels in model group were significantly increased compared with those in normal group (*p* < 0.05, *p* < 0.01). The TBIL and ALP levels in the PC group were reduced compared with those in model group, and no differences were observed when compared with those in the normal group. In contrast, the TBIL and ALP levels in three SS groups were higher than those in the model group. However, no significant differences were observed for the TBIL and ALP levels among the different SS doses.

In conclusion, oral SS administration demonstrated positive effects on serum liver function enzyme activity and liver function recovery, especially in the MSS and HSS groups.

### 3.9. Serum Lipid Levels

T2D is associated with other complications, such as cardiovascular disease. However, the incidence of cardiovascular complications can be reduced by monitoring serum lipid levels. To study the effects of SS on serum lipid levels, total cholesterol (CHOL), TG, HDL-C, LDL-C, LDH, and NEAF1 were measured (in Table 3).

CHOL refers to the total cholesterol level, including all lipoproteins in the blood. The CHOL level of model group was 2.61 ± 0.11 mmol/L (*p* < 0.01). After oral SS administration, the CHOL levels of three SS groups decreased significantly. The CHOL levels of the MSS and HSS groups decreased by approximately 25% compared with that of the model group. These results suggested that SS could effectively improve the serum CHOL levels in T2D rats.

TG is an important component of lipids, consisting of long-chain fatty acids and glycerol. The TG level of the model group significantly increased to 1.50 ± 0.20 mmol/L compared with that of normal group (*p* < 0.01). After four weeks of treatment, the TG level of the PC group was 13% lower than that of the model group. The TG levels of three SS groups displayed downward trends, which were all significantly reduced compared with that of the model group (*p* < 0.05, *p* < 0.01). Compared with the model group, the TG levels of the SS groups decreased by 27.3, 28, and 34.7%, and the TG level of the HSS group resembled that of the normal group.

Both HDL and LDL are closely related to cholesterol transport. The HDL-C levels of all three SS groups were significantly lower than those of the model and PC groups. The HSS group presented the best therapeutic effect, and the HDL-C value of this group was similar to that of the normal group. The LDL-C levels of three SS groups were lower than that of the model group, but higher than that of the PC group, and the LDL-C levels of the MSS and HSS group were similar to that for the normal group. These results indicated that SS could reduce LDL-C and HDL-C levels, with the strongest effect observed for the HSS group.

Abnormally increased LDH activity is an important indicator of myocardial infarction, acute or chronic hepatitis, and liver cancer. The serum non-esterified fatty acid (NEFA1) level is closely related to lipid metabolism, glucose metabolism, and endocrine function [30]. The NEFA1 level also increases due to diabetes. LDH and NEFA1 levels in the model group were significantly increased by approximately 50% compared with normal group, whereas the LDH level in the PC group significantly decreased (*p* < 0.05). The LDH and NEFA1 levels of three SS groups were significantly decreased (*p* < 0.01), with the strongest effect observed in the HSS group. These results suggested that SS was able to regulate the blood lipid metabolism of T2D rats by regulating serum lipid levels.

### 3.10. Liver Weights and Organ Coefficients

The liver is an important location for glucose, fat, and protein metabolism and is the primary site for insulin activity and catabolism. Diabetes can cause liver enlargement and abnormal liver function, which eventually leads to the development of liver lesions. To explore the effects of SS on the liver in T2D rats, the liver weights (wet tissue weight) and organ coefficients of each group were examined. The liver weight of the normal group was 16.38 ± 1.70 g, which was significantly reduced to 10.86 ± 1.43 g in the model group (*p* < 0.01). The liver quality of the PC group increased significantly compared with the model group, returning to a normal level. The liver quality of three SS groups also showed a slight upwards trend, but there was no significant difference in liver quality from model group. The organ coefficient of normal group was 3.25% ± 0.34%, which increased to 4.34% ± 0.54% in model group (*p* < 0.01). Although the organ coefficients for the PC group and three SS groups did not change significantly from model group, they all decreased to some degree, especially for the HSS group. These results indicated that high-dose sericin could improve liver hyperplasia and hypertrophy caused by diabetes.

### 3.11. Hepatic 8-Hydroxydeoxyguanosine Level

8-Hydroxydeoxyguanosine (8-OHdG) levels serve as a biomarker for DNA oxidative stress damage [31]. The 8-OHdG levels in the livers of all groups are presented in Figure 4C. The 8-OHdG level in normal group was 70.89 ± 0.61 ng/mg protein, and the 8-OHdG level in the model group significantly increased compared with that in the normal group (*p* < 0.01). After oral SS administration, the 8-OHdG levels in three SS groups significantly decreased in a dose-dependent manner compared with that in the model group (*p* < 0.01). The 8-OHdG levels were 7.2 ng/mg protein in the LSS group, which represented a 13.6% reduction compared with that in the model group, and the 8-OHdG levels resembled normal levels in the MSS group. The 8-OHdG levels in the HSS group were reduced by 20%, to 66.02 ± 5.45 ng/mg protein, which was even lower than the value observed for the normal group. These results indicated that oral SS administration had downregulated the abnormal increase of 8-OHdG level and may have increased the oxidative stress response in T2D rats.

### 3.12. Hepatic Glycogen

Hepatic glycogen plays an important role in the maintenance of glucose homeostasis [32]. Following a meal, the glucose balance is maintained by hepatic glycogen synthesis and storage. The synthesis and degradation of hepatic glycogen are primarily regulated by insulin and glucagon. As shown in Figure 4D, the hepatic glycogen level was 24.65 ± 1.36 ng/mL in normal group, whereas the level of model group was only 7.33 ± 1.25 ng/mL, which was less than 1/3 that in normal rats. The hepatic glycogen level of rats in the PC group increased significantly (by 150%), which indicated that orally administered metformin was able to effectively promote the synthesis and storage of liver glycogen. After oral SS administration, the hepatic glycogen levels of three SS groups increased significantly, demonstrating a positive and dose-dependent trend. The hepatic glycogen level of the LSS group was 14.73 ng/mL, which was twice that of the model group. The hepatic glycogen levels of both the MSS and HSS groups were higher than 20 ng/mL, which approached the level of normal rats. These results indicated that sericin could significantly regulate the synthesis and decomposition of hepatic glycogen in T2D rats.

### 3.13. Hepatic Antioxidant Capacity

Oxidative stress in the liver often leads to glucose metabolism disorders, and antioxidant enzymes can prevent oxidative stress damage by scavenging free radicals to maintain normal active oxygen levels. To examine the hepatic antioxidant capacity across the various experimental groups, GSH-PX, T-SOD, MDA, and T-AOC were measured, as shown in Table 4 [33].

GSH-PX not only reduces toxic peroxides into non-toxic hydroxyl compounds, but also decreases the formation of lipid peroxides, which enhances the body’s ability to resist oxidative stress damage [34]. The GSH-PX level of normal group was 1410 ± 21 U/mg protein. Compared with the normal group, the hepatic GSH-PX level of the model group was significantly reduced. The hepatic GSH-PX level of the PC group increased slightly. Compared to the model group, the GSH-PX level of the LSS group increased by 13.5%, and the GSH-PX levels of the MSS and HSS groups increased by 53.8 and 58.2%, respectively. The above results indicated that oral SS administration for four weeks could effectively restore the loss of hepatic GSH-PX levels in T2D rats.

SOD is an antioxidant enzyme that is widely found in organisms and is the primary molecule responsible for free radical scavenging [35]. SOD plays an extremely important role in reducing cell damage. The SOD level of the normal group was 103.83 U/mg protein, and the SOD level of the model group was significantly decreased compared with that of the normal group (*p* < 0.001). The SOD level of the PC, LSS, and MSS groups were reduced even more than that of the model group, whereas the SOD level of the HSS group increased significantly compared with that of the model group and approached the level observed in the normal group.

MDA is the oxidation product of lipid peroxidation reaction, in vivo, which causes the cross-linking and polymerization of biological macromolecules, denaturing biofilms and causing cell senescence or death [36]. The MDA level in normal group was 1.54 ± 0.46 nmol/mg protein, and the level in model group increased significantly compared with that in the normal group. Compared with that in the model group, the MDA level of rats in the PC group was significantly reduced *(p* < 0.05). The MDA level of the SS group decreased in a dose-dependent trend.

T-AOC reflects the total antioxidant level, comprising antioxidant substances and antioxidant enzymes in the body [37]. This study used the ABTS method to determine the T-AOC of the liver. The T-AOC level in the normal group was 128.8 ± 3.38 nmol/mg protein, and the T-AOC level of the model group was only 54.40 ± 4.19 nmol/mg protein, which was less than 50% that of the normal group (*p* < 0.01). Compared with the model group, the T-AOC level of the PC group significantly increased, and the T-AOC levels of three SS groups were all higher than that of model group. The T-AOC levels of these three SS groups were 218, 221, and 280% that of the model group. The T-AOC level of the HSS group was 18% higher than that of the normal group. Therefore, SS can significantly enhance the T-AOC enzyme activity in the liver of T2D rats.

In conclusion, these experimental results suggested that oral sericin administration could effectively improve antioxidant enzyme activity in T2D rats and eliminate the oxidative stress caused by STZ, especially in the HSS group. Sericin was able to effectively enhance antioxidant capacity by increasing GSH- PX, SOD, and T-AOC levels and reducing the MDA level.

### 3.14. Liver Pathological Tissue

**Figure 5:**
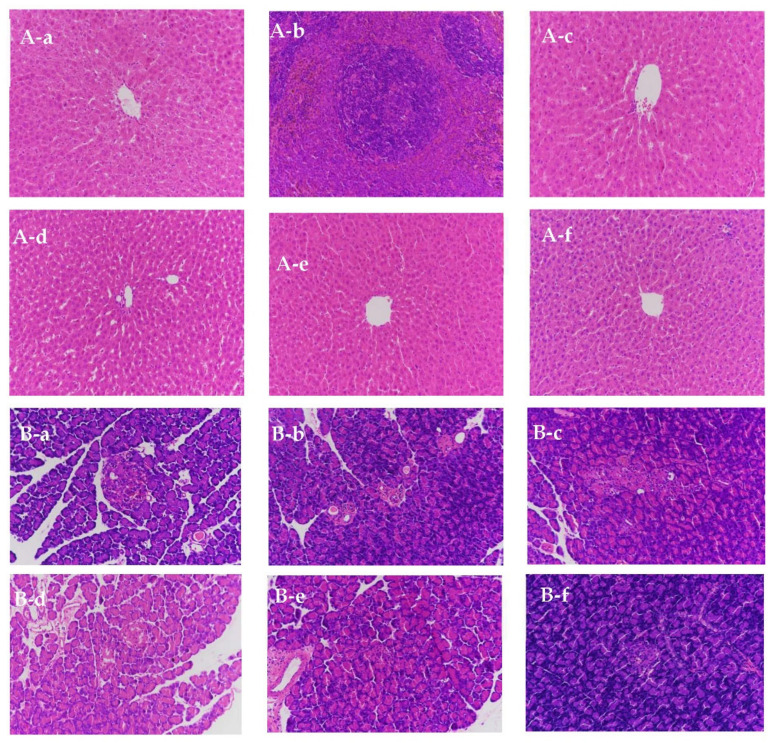
Effects of oral SS administration on HE staining in the liver (**A**) and pancreas tissues (**B**) of T2D rats (×100); **a**, normal group; **b**, diabetic model group; **c**, metformin administration; **d**, 1% SS (LSS); **e**, 2.5% SS (MSS); and **f**, 5% SS (HSS) administration.

The liver is an important organ in the body, which regulates blood glucose stability [38]. To study the effects of oral SS on liver injury recovery in T2D rats, pathological liver tissues were analyzed. The liver cells of normal group were arranged neatly, and the liver lobule structure was clear and complete, as shown in Figure 5A. The liver tissues of model rats were greatly changed, with reduced liver cell volumes, an extremely dense cell arrangement, and clear signs of inflammatory infiltration. Although some of the cells in the liver lobules of the PC group were ruptured, the whole liver lobule structure was clear, with no apparent vacuoles or inflammatory infiltrations. In three SS groups, the pathological liver tissues appeared to have recovered, to a certain extent. Although some apparent vacuoles and slight inflammation could be observed in the pathological liver slices of the LSS group, the overall liver lobule structure was clear. The abnormal pathological changes in the livers from the MSS and HSS group were obvious, the liver cells were in a good arrangement, the structure of the liver lobules was complete, and no apparent vacuole or inflammatory infiltration was observed, as shown in Figure 5(A-e,A-f). The pathological liver tissue examination of T2D rats showed that oral SS administration could effectively reduce liver damage by significantly reducing the occurrence of inflammation and infiltration, especially in the MSS and HSS groups.

### 3.15. Pancreatic Histopathological Sections

The pancreas is an important location for insulin secretion and is affected in T2D patients. To study the protection exerted by SS against pancreatic injury in T2D rats, pancreatic histopathological sections were observed and analyzed. The results are shown in Figure 5B. The pancreatic tissues from rats in normal group were intact, with normal morphology and islet cells arranged in order (Figure 5(B-a)). In the model group, the pancreatic structure was broken and incomplete. Several small and scattered pancreatic tissues can be observed in Figure 5(B-b). The number of pancreatic islet cells was reduced, cytoplasmic vacuolation could be observed in several cells, and inflammatory and fat infiltration occurred. As shown in Figure 5B-c, the structures of pancreatic cells in the PC group were irregular, and inflammation can be observed in the cell cytoplasm. The pancreatic structure became clearer and more complete in HE staining performed for three SS groups (Figure 5(B-e,B-f)). The pancreatic cells increased, with a dose-dependent effect. These results indicated that SS might facilitate the recovery of pancreatic damage caused by diabetes.

### 3.16. Pancreatic Immunofluorescence Staining

**Figure 6:**
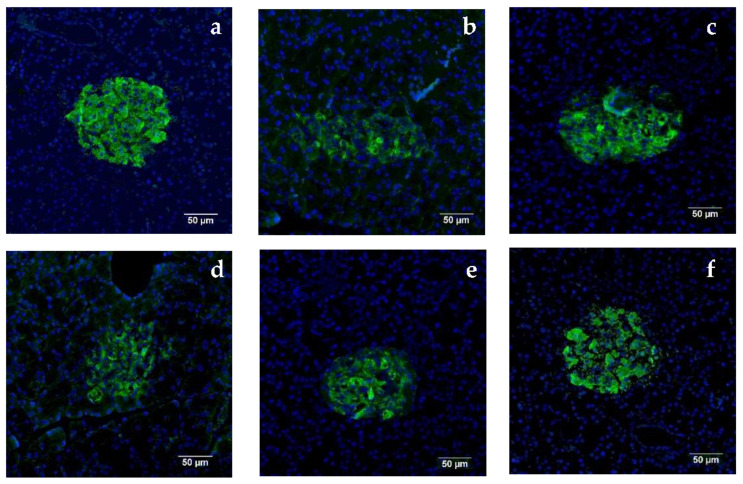
Effects of oral SS administration on pancreatic immunofluorescence staining in T2D rats (×400) (**a**), normal group; (**b**), diabetic model group; (**c**), metformin administration; (**d**), 1% SS(LSS); (**e**), 2.5% SS (MSS); and (**f**), 5% SS (HSS) administration.

To explain the effects of oral SS administration on the insulin expression levels of T2D rats, insulin immunofluorescence staining of the pancreas was performed. The results are shown in Figure 6, with green fluorescence indicating insulin-positive staining. The islet structure in the normal group was relatively complete and oval-shaped. Several islet β cells could be detected, and the insulin level was abundant in the normal group (a). In the model group (b), the structure of the rat pancreatic islets was loose and incomplete, with very weak green fluorescence intensity. Compared with the model group, the green fluorescence intensity of three SS groups appeared to recover, to different degrees. The fluorescence area expanded, with a dose-dependent trend. The insulin level gradually increased, the number of islets β cells gradually increased, and the pancreatic structures appeared more complete with increases in SS concentrations. SS treatment was supposed to repair the abnormal insulin secretion of pancreatic cells in T2D rats.

## 4. Discussion

T2D is also referred to as non-insulin-dependent diabetes. The pathogenesis of T2D is complicated, accompanied by hypertension and hyperlipidemia. Insulin resistance in surrounding tissues plays an important role in pathogenesis of T2D [26,39,40]. Currently, the primary treatment for T2D is mainly medication. Although some diabetes drugs can effectively control blood glucose levels, they are often associated with side effects, such as hypoglycemia, osteoporosis, or heart failure [41]. Biologically active peptides represent a potential alternative to diabetes drugs, associated with minimal side effects [42,43]. After oral SS administration, blood glucose levels were significantly reduced, the destruction of islet cells was reduced, and the liver and pancreas appeared to be protected from damage [44]. SS was mixed into feed powder at different concentrations and orally administered for four weeks to explore the hypoglycemic effect of oral SS in T2D rats.

The most typical feature of T2D is glucose metabolism disorder, which can cause oxidative stress and other reactions that activate various inflammatory factors and finally cause bodily dysfunction. The regulation of blood glucose levels is closely associated with the secretion and function of insulin. Therefore, controlling blood glucose levels and regulating insulin secretion are two important methods for treating T2D. After oral SS administration for four weeks, weight loss in each SS group was effectively relieved, and FBG was significantly reduced, especially in MSS and HSS groups, for which the blood glucose reduction rates were 62.3 and 67.1%, respectively. The observed decrease in GSP levels, which decreased during the second to fourth weeks of oral SS administration, was also dose-dependent. Serum insulin levels were significantly reduced, insulin resistance decreased, and insulin sensitivity increased, which indicated that sericin could enhance the effects of insulin on glucose absorption and utilization. Therefore, oral SS administration could improve glucose metabolism by reducing blood glucose levels, regulating insulin secretion, and relieving insulin resistance in T2D rats.

Lipid metabolism disorder is a clinical symptom of diabetes [22]. The test results for serum lipids showed that after the oral administration of sericin peptides, the levels of CHOL, TG, HDL, LDL, LDH, and NEAF1 in diabetic rats decreased, resulting in improved blood lipid levels.

Long-term high blood glucose levels in diabetes may cause liver enlargement, abnormal liver function, and even fatty liver and cirrhosis. Comparing the liver quality and organ coefficients of each group demonstrated that SS could improve the hyperplasia and hypertrophy of the liver caused by diabetes. The pathological liver sections showed that the liver cells in T2D rats were seriously damaged, featuring liver steatosis, inflammatory infiltration, and edema. The serum ALT, AST, and ALP levels were significantly increased, which also indicated liver damage. After oral SS administration, liver cells were tightly packed, inflammation was reduced, and the levels of liver functional enzymes, such as ALT and AST, also tended to be normal, which indicated that oral SS administration could effectively restore liver damage.

Oxidative stress damage often occurs during diabetes, especially in the liver [45]. The antioxidant defense protects the body from oxidative stress by scavenging free radicals [46]. T-AOC reflects the body’s total antioxidant capacity, whereas GSH-PX can reduce lipid peroxides and resist oxidative damage in vivo. SOD is an important enzyme that scavenges reactive oxygen species and protects liver tissue from oxidative stress. The levels of MDA and 8-OHdG reflect lipid peroxidation and cell damage in the liver, respectively. The results suggested that oral sericin administration could improve the activities of T-AOC and GSH-PX and reduce the activities of MDA and 8-OHdG, reducing oxidative stress damage in the liver. Hepatic glycogen levels play an important role in maintaining blood glucose stability. Many animal experiments have shown that the hepatic glycogen levels in T2D rats decrease and that metformin can increase glycogen synthesis and reduce hyperglycemia. The results in this study were similar to most previous studies and were also consistent with the results observed for sericin treatments in T2D mice in our earlier studies [47]. The hepatic glycogen levels of the model group decreased compared with normal mice, whereas that for the PC group increased significantly. After four weeks of oral SS, three SS groups gradually increased, with a dose-dependent effect, which suggested that oral SS could promote hepatic glycogen synthesis to improve the hyperglycemic state.

The primary pathological site of diabetes is the pancreas in diabetes. To improve the pancreas, the maintenance of pancreatic function and reduced pancreatic β-cell apoptosis is necessary. In this experiment, HE and immunofluorescence staining were used to observe the pancreatic tissue structures of each group. After oral SS administration, the islet structure was relatively complete, the destruction of islets was greatly reduced, and the number of β-cells increased. In summary, oral SS may provide protection for islet β cells and could improve the structure and function of islets in T2D rats.

The experimental results obtained from the above are also consistent with our previous results obtained with STZ-induced type 2 diabetic mice [48] and the results by other authors [49,50]. After oral administration of degraded sericin peptide, the antioxidant capacity of diabetic rats was significantly improved. It evidently slows down the damage of oxidative stress and inflammation caused by STZ to rats, especially the liver and pancreas, and ultimately leads to partial repairing of their functions and results in a significant decrease in blood glucose levels in the body. In the future, we will use Western blotting and other methods to continue to explore the possible mechanism of oral sericin to reduce blood sugar in diabetic rats through signal pathways such as sugar metabolism, fat metabolism, and insulin synthesis.

## 5. Conclusions

After oral SS administration for four weeks, the rats in three oral sericin groups recovered following the induction of T2D rats. Weight loss was alleviated in diabetic rats that received middle and high doses of sericin, the FBG reduction rate was greater than 35%, and abnormal glucose tolerance and insulin tolerance were significantly improved. The serum insulin level was reduced, insulin resistance was reduced, and insulin sensitivity was enhanced. The serum GSP levels were significantly reduced. To some extent, sericin peptides exerted hypoglycemic effects. In addition, sericin peptides showed positive effects on the restoration of serum liver function enzymatic activities, which can reduce CHOL, TG, HDL, LDL, LDH, and NEAF1. In addition, sericin peptides could also improve the hyperplasia and hypertrophy of the liver. Sericin peptides were able to reduce oxidative damage, balance the synthesis and decomposition of glycogen, and partly restore the antioxidant capacity in the liver of T2D rats. Sericin peptides could also effectively reduce inflammatory infiltration, restore damaged liver cells, and repair liver damage. Sericin peptides also affected the recovery of pancreatic damage in a dose-dependent manner. The insulin immunofluorescence staining assay results showed that sericin could improve abnormal pancreatic insulin secretion in diabetic rats. Therefore, sericin recovered from industrial silk waste has potential uses as a health food additive and biomedicine, with hypoglycemic function during T2D.

## Figures and Tables

**Table 1:** Effects of oral SS administration on HOMA-IR and ISI values in T2D rats.

Groups	HOMA-IR	ISI
Normal	4.99 ± 0.03	−4.72 ± −0.26
Model	33.09 ± 0.04 ***	−6.61 ± −0.07 ***
PC	11.93 ± 0.03 ***^,###^	−5.59 ± −0.24 **^,###^
LSS	17.17 ± 0.02 ***^,###^	−5.96 ± −0.21 ***^,###^
MSS	10.25 ± 0.02 ^###^	−5.44 ± 0.14 ^###^
HSS	11.38 ± 0.04 ***^,###^	−5.54 ± −0.09 **^,###^

Normal: normal group; Model: diabetic model group; PC: 0.5% metformin administration; LSS, MSS, and HSS: 1, 2.5, and 5% SS administration; *n* = 3, ** *p* < 0.01 & *** *p* < 0.001, vs. normal group; ^###^ *p* < 0.001, vs. diabetic model group.

**Table 2:** Effects of oral SS administration on serum liver functional enzymes in T2D rats.

Groups	ALT U/L	AST U/L	TP G/L	ALB G/L	TBIL Umol/L	ALP G/L
Normal	30.00 ± 3.00	119.60 ± 2.08	37.07 ± 3.51	34.76 ± 0.71	0.50 ± 0.17	0.29 ± 0.02
Model	39.67 ± 3.06 *	187.67 ± 3.79 **^,##^	40.96 ± 0.56 *	42.07 ± 3.05 **	1.47 ± 0.32 *	0.49 ± 0.06 **
PC	38.33 ± 1.53 *	97.33 ± 2.52 **^,##^	41.10 ± 1.10 *	39.00 ± 0.85 **	0.33 ± 0.11 *^##^	0.22 ± 0.02 *^,##^
LSS	43.33 ± 5.69 **	109.00 ± 2.65 **^,##^	42.06 ± 1.36 *	36.83 ± 2.58	0.73 ± 0.05 ^#^	0.37 ± 0.08
MSS	35.67 ± 1.15 *	85.67 ± 3.21 **^,##^	36.87 ± 3.39 ^##^	35.80 ± 1.83 ^#^	0.80 ± 0.26 ^#^	0.36 ± 0.02 *^,#^
HSS	33.67 ± 3.21 ^#^	87.33 ± 0.58 **^,##^	38.93 ± 2.06 ^##^	37.56 ± 0.75 ^#^	0.80 ± 0.30 *	0.38 ± 0.01 **^,#^

ALT: alanine aminotransferase; AST: aspartate aminotransferase; TP: total protein; ALB: albumin; TBIL: total bilirubin; ALP: alkaline phosphatase; Normal: normal group; Model: diabetic model group; PC: 0.5% metformin administration; LSS, MSS, and HSS: 1%, 2.5%, and 5% SS administration; *n* = 3, * *p* < 0.05 & ** *p* < 0.01, vs. normal group; ^#^ *p* < 0.05 & ^##^ *p* < 0.01, vs. diabetic model group.

**Table 3:** Effect of oral SS administration on serum lipid levels in T2D rats.

Groups	CHOL mmol/L	TG mmol/L	HDL mmol/L	LDL mmol/L	LDH mmol/L	NEFA1 mmol/L
Normal	1.75 ± 0.11	0.93 ± 0.05	1.05 ± 0.02	0.44 ± 0.04	1.02 ± 0.08	0.69 ± 0.03
Model	2.61 ± 0.11 **	1.50 ± 0.20 **	1.50 ± 0.12 **	0.62 ± 0.15 *	1.65 ± 0.20 *	0.95 ± 0.02 **
PC	2.33 ± 0.31 *	1.31 ± 0.35 **	1.39 ± 0.08 **	0.38 ± 0.12 ^#^	1.16 ± 0.10 ^#^	1.10 ± 0.01 **
LSS	2.32 ± 0.37	1.09 ± 0.07 *^,##^	1.24 ± 0.03 *	0.49 ± 0.05	1.26 ± 0.01 *^,#^	0.55 ± 0.02 ^#^
MSS	1.97 ± 0.12 ^##^	1.05 ± 0.08 **^,#^	1.28 ± 0.06	0.42 ± 0.01 ^#^	1.09 ± 0.01 ^##^	0.44 ± 0.03 **^,##^
HSS	1.93 ± 0.15 ^##^	0.98 ± 0.05 ^#^	1.09 ± 0.05	0.42 ± 0.03 ^#^	1.18 ± 0.01 ^#^	0.43 ± 0.02 **^,##^

CHOL: total cholesterol; TG: triglycerides; HDL: high-density lipoprotein; LDL: low-density lipoprotein; LDH: lactose dehydrogenase; NEFA: non-esterified fatty acids; Normal: normal group; Model: diabetic model group; PC: metformin administration; LSS, MSS, and HSS: 1, 2.5, and 5% SS administration. *n* = 3, * *p* < 0.05 & ** *p* < 0.01, vs. normal group; ^#^ *p* < 0.05 & ^##^ *p* < 0.01, vs. diabetic model group.

**Table 4:** Effects of oral SS administration on the antioxidant activities in the liver of T2D rats.

Groups	GSH-PX U/mgprot	SOD U/mgprot	MDA nmol/mgprot	T-AOC nmol/mgprot
Normal	1410 ± 21	103.83 ± 0.95	1.54 ± 0.46	128.30 ± 3.38
Model	913 ± 35 **	90.71 ± 0.86 **	2.18 ± 0.05 **	54.40 ± 4.19 **
PC	1158 ± 18 **^,##^	79.38 ± 0.62 **^,^^##^	2.03 ± 0.11 **^,^^##^	113.71 ± 0.01 **^,^^##^
1% SS	1037 ± 60 *	77.26 ± 1.32 **^,^^#^	3.14 ± 0.27 **^,^^##^	118.73 ± 5.97 ^##^
2.5% SS	1404 ± 24 ^##^	76.31 ± 6.70 *^,^^##^	2.04 ± 0.23 **	120.06 ± 8.34 ^##^
5% SS	1444 ± 24 ^##^	99.22 ± 1.82 *^,^^##^	1.71 ± 0.07 *^,^^##^	152.43 ± 2.27 ^##^

GSH-PX: glutathione peroxidase; SOD: superoxide dismutase; MDA: malondialdehyde; T-AOC: total antioxidant concentration; Normal: normal group; Model: diabetic model group; PC: 0.5% metformin administration; LSS, MSS, and HSS: 1, 2.5, and 5% SS administration; * *p* < 0.05 & ** *p* < 0.01, vs. normal group; *n* = 3, ^##^ *p* < 0.01, vs. diabetic model group.

## Data Availability

Code and material; The datasets used and/or analyzed during the current study as well as analysis scripts are available from the corresponding author on reasonable request.

## Abbreviations

8-OHdG	8-hydroxydeoxyguanosine
ALB	albumin
ALP	alkaline phosphatase
ALT	alanine aminotransferase
AST	aspartate aminotransferase
AUC	area under the blood glucose curve
BCA	bicinchoninic acid
BW	body weight
CHOL	total cholesterol
EDTA	Ethylenediaminetetraacetic acid
FBG	fasting blood glucose
GSH-PX	glutathione peroxidase
GSPs	glycated serum proteins
HDL-C	high-density lipoprotein cholesterol
HE	hematoxylin-eosin
HOMA-IR	Homeostatic Model Assessment for Insulin Resistance
HSS	high dose sericin
ISI	insulin sensitivity index
ITT	insulin tolerance test
LDH	lactate dehydrogenase
LDL-C	low-density lipoprotein cholesterol
LSS	low dose sericin
MDA	malondialdehyde
MSS	medium dose sericin
NEAF	non-esterified free fatty acids
OGTT	oral glucose tolerance test
PBS	phosphate-buffered saline
PC	positive control
SD	Sprague-Dawley rat
SPF	specific-pathogen-free
SS	silk sericin
STZ	streptozocin
T2D	type 2 diabetic
T-AOC	total antioxidant capacity
TBIL	total bilirubin
TG	triglycerides
TP	total protein
T-SOD	total superoxide dismutase

## References

[1] Zhang Y.-Q. (2002). Applications of natural silk protein sericin in biomaterials. Biotechnol. Adv..

[2] Lamboni L., Gauthier M., Yang G., Wang Q. (2015). Silk sericin: A versatile material for tissue engineering and drug delivery. Biotechnol. Adv..

[3] Cao T.-T., Zhang Y.-Q. (2016). Processing and characterization of silk sericin from Bombyx mori and its application in biomaterials and biomedicines. Mater. Sci. Eng. C.

[4] Capar G., Aygün S., Gecit M. (2008). Treatment of silk production wastewaters by membrane processes for sericin recovery. J. Membr. Sci..

[5] Cao T.-T., Zhou Z.-Z., Zhang Y.-Q. (2014). Processing of β-Glucosidase–Silk Fibroin Nanoparticle Bioconjugates and Their Characteristics. Appl. Biochem. Biotechnol..

[6] Khan M.R., Tsukada M., Gotoh Y., Morikawa H., Freddi G., Shiozaki H. (2010). Physical properties and dyeability of silk fibers degummed with citric acid. Bioresour. Technol..

[7] Arami M., Rahimi S., Mivehie L., Mazaheri F., Mahmoodi N.M. (2007). Degumming of Persian silk with mixed proteolytic enzymes. J. Appl. Polym. Sci..

[8] Yun H., Oh H., Kim M.K., Kwaka H.W., Leea J.Y., Umc I.C., Vootlad S.K., Leea K.H. (2013). Extraction conditions of Antheraea mylitta sericin with high yields and minimum molecular weight degradation. Int. J. Biol. Macromol..

[9] He H., Cai R., Wang Y., Tao G., Guo P., Zuo H., Chen L., Liu X., Zhao P., Xia Q. (2017). Preparation and characterization of silk sericin/PVA blend film with silver nanoparticles for potential antimicrobial application. Int. J. Biol. Macromol..

[10] Zhao Z.-L., Zhang Y.-Q. (2020). Greener degumming production of layered sericin peptides from a silkworm cocoon and their physicochemical characteristics and bioactivities in vitro. J. Clean. Prod..

[11] Zhao Z.-L., Li W.-W., Wang F., Zhang Y.-Q. (2018). Using of hydrated lime water as a novel degumming agent of silk and sericin recycling from wastewater. J. Clean. Prod..

[12] Dash R., Acharya C., Bindu P., Kundu S.C. (2008). Antioxidant potential of silk protein sericin against hydrogen peroxide-induced oxidative stress in skin fibroblasts. BMB Rep..

[13] Kato N., Sato S., Yamanaka A., Yamada H., Fuwa N., Nomura M. (1998). Silk Protein, Sericin, Inhibits Lipid Peroxidation and Tyrosinase Activity. Biosci. Biotechnol. Biochem..

[14] Aramwit P., Kanokpanont S., De-Eknamkul W., Srichana T. (2009). Monitoring of inflammatory mediators induced by silk sericin. J. Biosci. Bioeng..

[15] Lamboni L., Li Y., Liu J. (2016). Silk Sericin-Functionalized Bacterial Cellulose as a Potential Wound-Healing Biomaterial. Biomacromolecules.

[16] Limpeanchob N., Trisat K., Duangjai A., Tiyaboonchai W., Pongcharoen S., Sutheerawattananonda M. (2010). Sericin Reduces Serum Cholesterol in Rats and Cholesterol Uptake into Caco-2 Cells. J. Agric. Food Chem..

[17] Seo C.-W., Um I.C., Rico C.W., Kang M.Y. (2011). Antihyperlipidemic and Body Fat-Lowering Effects of Silk Proteins with Different Fibroin/Sericin Compositions in Mice Fed with High Fat Diet. J. Agric. Food Chem..

[18] Furuya S., Tabata T., Mitoma J., Yamada K., Yamasaki M., Makino A., Yamamoto T., Watanabe M., Kano M., Hirabayashi Y. (2000). L-Serine and glycine serve as major astroglia-derived trophic factors for cerebellar Purkinje neurons. Proc. Natl. Acad. Sci. USA.

[19] Lee H.J., Lee H.-S., Choi J.W., Ra K.S., Kim J.-M., Suh H.J. (2011). Novel Tripeptides with α-Glucosidase Inhibitory Activity Isolated from Silk Cocoon Hydrolysate. J. Agric. Food Chem..

[20] Okazaki Y., Kakehi S., Xu Y., Tsujimoto K., Sasaki M., Ogawa H., Kato N. (2010). Consumption of Sericin Reduces Serum Lipids, Ameliorates Glucose Tolerance and Elevates Serum Adiponectin in Rats Fed a High-Fat Diet. Biosci. Biotechnol. Biochem..

[21] Song C., Liu D., Yang S., Cheng L., Xing E., Chen Z. (2018). Sericin enhances the insulin-PI3K/AKT signaling pathway in the liver of a type 2 diabetes rat model. Exp. Ther. Med..

[22] Chen Z., He Y., Fu W. (2011). Effects of sericin on heme oxygenase-1 expression in the hippocampus and cerebral cortex of type 2 diabetes mellitus rats. Neural Regen. Res..

[23] Liu D., Liu M., Wang X., Zhang Y., Yang S., Cheng L., Chen Z. (2015). Effects of sericin on TNF-α and HNF-4α expressions in liver tissue of rats with type 2 diabetes mellitus. J. Jilin Univ..

[24] Xing T., Yang M. (2015). Prospects of Silk Sericin Membranes Fabricated with Tyrosinase. J. Fiber Bioeng. Inform..

[25] Chen Z., Yang S., He Y., Song C., Liu Y.P. (2013). Effect of sericin on diabetic hippocampal growth hormone/insulin-like growth factor 1 axis. Neural Regen. Res..

[26] Ampawong S., Isarangkul D., Aramwit P. (2016). Sericin ameliorated dysmorphic mitochondria in high-cholesterol diet/streptozotocin rat by antioxidative property. Exp. Biol. Med..

[27] Schleicher R., Wieland O.H. (1989). Protein glycation: Measurement and clinical relevance. J. Clin. Chem. Clin. Biochem..

[28] Tong X., Xu J., Lian F., Yu X., Zhao Y., Xu L., Zhang M., Zhao X., Shen J., Wu S. (2018). Structural Alteration of Gut Microbiota during the Amelioration of Human Type 2 Diabetes with Hyperlipidemia by Metformin and a Traditional Chinese Herbal Formula: A Multicenter, Randomized, Open Label Clinical Trial. mBio.

[29] Shimobayashi M., Albert V., Wölnerhanssen B., Frei I.C., Weissenberger D., Meyer-Gerspach A.C., Clement N., Moes S., Colombi M., Meier J.A. (2018). Insulin resistance causes inflammation in adipose tissue. J. Clin. Investig..

[30] Huang L.P., Zhou R., Meng X.F., Yu R.Y., Sun J.N. (2010). Effect of cold property Chinese medicine radix scutellariae on energy metabolism of rats. China J. Chin. Mater. Med..

[31] Ma D., Zhu W., Hu S., Yu X., Yang Y. (2013). Association between oxidative stress and telomere length in Type 1 and Type 2 diabetic patients. J. Endocrinol. Investig..

[32] Sullivan T.J., Miao Z., Zhao B.N., Ertl L.S., Wang Y., Krasinski A., Walters M.J., Powers J.P., Dairaghi D.J., Baumgart T. (2013). Experimental evidence for the use of CCR2 antagonists in the treatment of type 2 diabetes. Metabolism.

[33] Yin X.-L., Liu H.-Y., Zhang Y.-Q. (2017). Mulberry branch bark powder significantly improves hyperglycemia and regulates insulin secretion in type II diabetic mice. Food Nutr. Res..

[34] Jotic A., Sternic N.C., Kostic V.S., Lalic K., Miličić T., Mijajlovic M., Lukic L., Civcic M., Colak E., Macesic M. (2013). Type 2 Diabetic Patients with Ischemic Stroke: Decreased Insulin Sensitivity and Decreases in Antioxidant Enzyme Activity Are Related to Different Stroke Subtypes. Int. J. Endocrinol..

[35] De Wet H., Levitt N., Tipping B. (2007). Executive cognitive impairment detected by simple bedside testing is associated with poor glycaemic control in type 2 diabetes. S. Afr. Med. J..

[36] Zhao J.-G., Wang H.-Y., Wei Z.-G., Zhang Y.-Q. (2019). Therapeutic effects of ethanolic extract from the green cocoon shell of silkworm Bombyx mori on type 2 diabetic mice and its hypoglycaemic mechanism. Toxicol. Res..

[37] Hedman K., Zilmer M., Sundström J., Lind L., Ingelsson E. (2016). DNA methylation patterns associated with oxidative stress in an ageing population. BMC Med. Genom..

[38] Islam S., Rahman S., Haque T., Sumon A.H., Ahmed A.M., Ali N. (2020). Prevalence of elevated liver enzymes and its association with type 2 diabetes: A cross-sectional study in Bangladeshi adults. Endocrinol. Diabetes Metab..

[39] Gaster B., Hirsch I.B. (1998). The effects of improved glycemic control on complications in type 2 diabetes. Arch. Intern. Med..

[40] Greenfield J.R., Campbell L.V. (2006). Relationship between inflammation, insulin resistance and type 2 diabetes: ‘Cause or effect’?. Curr. Diabetes Rev..

[41] Mahler R.J., Adler M.L. (1999). Type 2 Diabetes Mellitus: Update on Diagnosis, Pathophysiology, and Treatment. J. Clin. Endocrinol. Metab..

[42] Kalita D., Holm D.G., LaBarbera D.V., Petrash J.M., Jayanty S.S. (2018). Inhibition of α-glucosidase, α-amylase, and aldose reductase by potato polyphenolic compounds. PLoS ONE.

[43] Shaji J., Patole V. (2008). Protein and peptide drug delivery: Oral approaches. Indian J. Pharm. Sci..

[44] Li C., Eunice C.Y. (2015). Bioactive peptides and protein hydrolysates: Research trends and challenges for application as nutraceuticals and functional food ingredients. Curr. Opin. Food Sci..

[45] Sedlak L., Wojnar W., Zych M., Wyględowska-Promieńska D., Mrukwa-Kominek E., Kaczmarczyk-Sedlak I. (2018). Effect of Resveratrol, a Dietary-Derived Polyphenol, on the Oxidative Stress and Polyol Pathway in the Lens of Rats with Streptozotocin-Induced Diabetes. Nutrients.

[46] Sheweita S.A., Mashaly S., Newairy A.A., Abdou H., Eweda S.M. (2016). Changes in Oxidative Stress and Antioxidant Enzyme Activities in Streptozotocin-Induced Diabetes Mellitus in Rats: Role ofAlhagi maurorumExtracts. Oxidative Med. Cell. Longev..

[47] Ondreicka R., Beno I., Cerna O., Staruchova M., Volkvova K., Bobek P., Tatara M. (1998). Relation between levels of vitamins C, E, A and beta-carotene and activity of antioxidant enzymes in the blood. Bratisl. Lek. Listy.

[48] Dong X., Zhao S.-X., Yin X.-L., Wang H.-Y., Wei Z.-G., Zhang Y.-Q. (2019). Silk sericin has significantly hypoglycaemic effect in type 2 diabetic mice via anti-oxidation and anti-inflammation. Int. J. Biol. Macromol..

[49] Deori M., Devi D., Kumari S., Hazarika A., Kalita H., Sarma R., Devi R. (2016). Antioxidant Effect of Sericin in Brain and Peripheral Tissues of Oxidative Stress Induced Hypercholesterolemic Rats. Front. Pharmacol..

[50] Mohammadi A.B., Torbati M., Farajdokht F., Sadigh-Eteghad S., Fazljou S.M.B., Vatandoust S.M., Golzari S.E., Mahmoudi J. (2019). Sericin alleviates restraint stress induced depressive- and anxiety-like behaviors via modulation of oxidative stress, neuroinflammation and apoptosis in the prefrontal cortex and hippocampus. Brain Res..

